# Department of Canine, Feline, and Avian Medicine and Surgery

**Published:** 1902-07

**Authors:** Cecil French

**Affiliations:** D.V.S. (McGill and Munich), Washington, D. C.


### DEPARTMENT OF CANINE, FELINE, AND AVIAN MEDICINE AND SURGERY.

By Cecil French, D.V.S. (McGill and Munich),
WASHINGTON, D. C.

Report on the Death of a Hyena at the National Zoölogical Park. The animal in question was a female, and, judging from its dentition, which was of recent completion, aged about eight or ten months. I saw it on the morning of May 24th, and found it lying prostrate on its side and apparently unconscious. Its pulsations were then about 100, and there was no appreciable fever present. The iris responded normally to light rays. Auscultation of the lungs and heart with the aid of a phonendoscope disclosed nothing abnormal. The general condition suggested cerebral compression. The history of the case obtained from the attendant keepers was as follows: The animal, in company with a mate of the same age, had arrived at the Park about a month previously in very poor condition, but had improved considerably under a bountiful diet of chicken, pigeon, etc., including bones and viscera.

About the 18th of May the animal began failing, and continued to do so untill the early morning of the 23d, when it was observed to move backward away from the bars of its cage and fall on the floor. After that it did not again rise on its four feet, but if propped up on its front legs would remain in that position for a few minutes at a time. Death occurred the night of the 24th–25th, and the body was conveyed to the taxidermist's laboratory at the Smithsonian Institution, where I made a necropsy at 1 o'clock on the 25th, the animal then having been dead some hours.

Briefly, the macroscopical lesions present were as follows:

*Thoracic Cavity.* It was here that the most pronounced lesions had taken place. The pleural and pericardial sacs contained a considerable quantity of clear sanguineous fluid, and the endocardium was highly injected, being almost black in places. Both lungs were greatly congested, the bronchioles containing mucus in profusion. In no part of the body examined was the blood coagulated.

*Abdominal Cavity.* The liver and kidneys were congested, but the spleen appeared normal. The stomach and intestines were empty, the former showing a few faint ecchymoses of the mucosa, the latter being apparently normal.

*Head.* On opening the cranium a quantity of subdural fluid was found present, and the superficial vessels of the brain were injected. The brain itself was exceedingly soft, falling to pieces during the process of removing it.

A noticeable feature about the reproductive organs of the hyena, and which were evidently normal, was the mammillate ovary possessed by this animal.

I removed portions of the liver, lungs, and pericardial fluid for microscopical examination.

From a bacteriological standpoint this case has proved to be of more than ordinary interest, for it has afforded a well-marked example of the capacity which certain micro-organisms possess of undergoing alterations in shape when cultivated on artificial media.

There were present in the liver, lungs, and pericardial fluid two micro-organisms. One occurred in chains and closely resembled the anthrax bacillus, but was much larger than the latter. It held Gram's stain. As this bacterium disappeared in the subsequent cultivation, it must be assumed that it was an anaërobic bacillus. The other micro-organism appeared as a coccus or extremely short bacillus, singly and in pairs. This caused us to suspect that we might be dealing with a member of the hemorrhagic septicæmia group of micro-organisms, and, inasmuch as the dead animal had been fed upon whole uncooked chicken, the prospect seemed bright of being able to trace its death to this source. But subsequent developments showed this hypothesis to be untenable.

Transfers from single colonies on agar plate, which had been obtained in pure form from the lung, to agar slants showed a distinct bacillus form. Grown on blood serum and potato it appeared about the size of the colon bacillus, and as the transfers fermented the three sugars and produced indol in Dunham's solution, and gave a moderate motility, it was, in fact, this micro-organism. Moreover, its pathogenesis further proved this, for 1 c.c. and 2 c.c. of a bouillon culture were injected into a young rabbit and a young guinea-pig intraperitoneally, respectively, and produced death by toxæmia within three hours. I recovered the micro-organism from the heart blood of these animals immediately after death. Two guinea-pigs and a rabbit were further inoculated subcutaneously with $\frac{1}{5}$ c.c. each, and developed local abscesses. A young chicken was fed on bouillon and agar cultures, but without any effect on its health.

With regard to the change in shape exhibited by this bacterium, the phenomenon has been recorded within the last few years by several bacteriologists, notably Professor Adami, of McGill University, in conjunction with Drs. Abbott and Nicholson.[1]

These gentlemen made the observation that the colon bacillus when injected into the circulation is quickly ingested by the endothelial cells in the liver, thereby being broken up into shorter lengths to resemble diplococci such as appeared in the tissues of our hyena. Furthermore, certain body fluids, such as ascitic and peritoneal fluids, sown with the typical form of the bacillus yielded the short diplococcoid growths under prolonged action. This modified form is non-motile and without the power to ferment the sugars and to develop the indol reaction.

Ordinary nutritive media also show the modified form quite frequently.

What caused the death of the hyena it is difficult to say. The anaërobic bacillus alluded to may have been responsible; but, on the other hand, its presence in the tissues may be accounted for by its possible entrance after death.

That the colon bacillus had something to do with it seems more likely, but our present limited knowledge of the pathogenic capacity of this micro-organism on the carnivora forbids a positive conclusion. It is generally believed that some initial lesion is necessary for its entrance into the system in such numbers as to produce profound effect. Drs. Reed and Carroll, of the Army Medical Museum, were able to produce hemorrhagic lesions in the stomach and intestines in dogs by intravenous injection of a member of the colon group, the bacillus X of Sternberg.[2] Some years ago a case came under my observation in which the animal, an aged bitch, died while suffering from acute inflammation, and ulceration of the intestines and extreme tape-worm infestation, together with focal necrosis of the liver.[3] On this occasion I also observed the presence of a micro-organism of diplococcoid form, and which,

1 Journal of Experimental Medicine, 1899, iv. p. 349.
2 Ibid., 1900, v. No. 3.
3 Journal of Comparative Medicine and Veterinary Archives, 1900, xxi. p. 350.

cultivated by Dr. Adami, proved to be the colon bacillus, and which seemed to be present in pure form, as none of the ordinary bacteria could be recognized. In this case an initial lesion might well have been produced by the burrowing proclivity of the tapeworm.

But in the hyena there was no evidence of any lesion in the alimentary canal, the only tract by which the colon bacillus would have been likely to enter, unless it had sustained wounding at some earlier date by the passage of chicken-bones along it.

On the other hand, the prostration of the animal may have been the result of pathological changes taking place within the cranial cavity, the colon bacillus invading the circulation and producing the lesions within the thoracic cavity during the last hours of life while the vitality of the animal was reduced. There is some reason for considering this hypothesis because of the absence of the characteristic signs of the presence of fluid when I examined the thoracic cavity with the phonendoscope, an instrument with which the faintest sound may be detected.

The cause of death must, therefore, remain undetermined, with the probability that the colon bacillus figured in the matter.

---

Cutaneous Lymphadenoma in a Cow. Liénaux reports an observation of a cow, three years old and cachectic. The skin, excepting at the extremity of the legs, was covered with circular elevations 4 to 5 centimetres in diameter, slightly convex and smooth. These tumors were flat, slightly elastic, non-inflammatory, and covered with epidermis which was healthy, smooth or slightly scaly, and deprived of hair.

The nature of these tumors could not be recognized excepting for a tumefaction of parotid, sublingual, retropharyngeal, prescapular, precrural and supramammary glands. The diagnosis of lymphadenoma was proved by a histological examination of the tumors and counting of the blood corpuscles.

At the necropsy the deep lymphatic glands were also affected; the spleen and Peyer's patches were not hypertrophied.

The localization of lymphadenoma in the cow is a new pathological phenomenon and very rare in veterinary medicine. In human medicine it is quite common.—*Rec. de Med. Vet.*

